# Neuroendocrine carcinoma of esophageal and gastric cardia: clinicopathologic and immunohistochemistry study of 80 cases

**DOI:** 10.18632/oncotarget.23610

**Published:** 2017-12-22

**Authors:** Liangli Hong, Ying Zhang, Zhaoyong Liu

**Affiliations:** ^1^ Department of Pathology, First Affiliated Hospital of Shantou University Medical College, Shantou, Guangdong, China; ^2^ Department of Pathology, Shantou University Medical College, Shantou, Guangdong, China; ^3^ Department of Orthopedics, First Affiliated Hospital of Shantou University Medical College, Shantou, Guangdong, China

**Keywords:** neuroendocrine tumor, esophageal, gastric cardia, prognostic

## Abstract

Neuroendocrine carcinoma (NEC) of the esophagus and gastric cardia is a rare tumor, and the Chaoshan region has one of the highest incidences of esophageal and gastric cardia cancer (GCC) worldwide. The aim of this study was to characterize the clinicopathologic features of esophageal NEC (*n* = 67) and gastric cardia NEC (*n* = 13) cases identified over a 9-year period in the Chaoshan region. Esophageal NECs were either purely NEC (*n* = 47) or mixed with squamous cell carcinoma or adenocarcinoma (*n* = 20). For GCC; pure NEC was found in 5 cases, whereas 8 cases were mixed with adenocarcinomas. The majority of esophageal and gastric cardia NECs was of the small cell type, and 24/67 esophageal and 5/13 gastric cardia patients were found with lymph node metastasis. Immunohistochemistry was performed in all cases, and positive staining for synaptophysin (Syn) was found for all cases, with half the esophageal NEC cases being also chromogranin A (CgA)-positive. In the multivariate Cox regression model, lymph node and further metastasis were independent prognostic factors for esophageal NEC. Our study revealed the clinicopathological features of esophageal and gastric cardia NECs in the Chaoshan region and found mixed NECs patients may have a better prognosis than pure NECs patients, which may provide therapeutic clue for treating this rare tumor.

## INTRODUCTION

Neuroendocrine tumors(NETs) are defined as neoplasms that exhibit neuroendocrine phenotypes, such as production of neuropeptides, large dense-core secretory vesicles, and a lack of neural structures [1]. According to the World Health Organization (WHO) classification scheme in 2010, NETs can bedivided into three main categories: well-differentiated (low-grade), which is benign, medium-differentiated (intermediate-grade) with low-grade malignant behavior, and poorly-differentiated (high grade), which can itself be divided into large cell and small cell neuroendocrine carcinomas (NECs) [2]. Additionally, adenocarcinomas that contain neuroendocrine cells (exceeding at least 30% of all tumor cells) mixed with non-endocrine components (usually adenocarcinoma-like structures) are classified asmixed adenoneuroendocrine carcinomas (MANECs) [3]. NEC, a malignant type of NET, has been reported in pancreatic, breast, lung and bronchial cancers [4–6].

Esophageal NEC is a rare esophageal cancer consisting of neuroendocrine components, and comprises 0.5–5.9% of all esophageal malignancies. During the past 20 years, a limited number of cases has been reported in the literature.NECs appears to confer a worse prognosis than other malignant esophageal cancers, probably due to its poor differentiation and lymphovascular invasion. However, the clinicopathologic characteristics of NECs have not been fully described.

The gastric cardia is a very narrow area between the esophagus and gastric fundus. This specialized mucosa contains a mixture of squamous and glandular epithelium [7]. The gastric cardia mucosa is gastric in origin, and strong evidence shows that the gastric cardia contains numerous endocrine cells, which are mainly located on the surfaces of adjacent mucosal or parietal cells [8]. Owing to its unique location and biological features, gastric cardia cancer is mainly divided into two distinct types, one type being located in the distal stomach as a consequence of atrophic gastritis, and the other type resembling esophageal adenocarcinoma that may be a consequence of short-segment gastro-esophageal reflux disease [7]. However, NECs in the gastric cardia are rare, representing only 0.04–7.6% of the gastrointestinal NECs that have been reported [9].

The Chaoshan area has the highest incidence of esophageal cancer and gastric cardia cancer worldwide, with the age-standardized incidence rates for esophageal and gastric cardia cancer in the Chaoshan area respectively being 100/100,000 and 34.81/100,000, which are much higher than the worldwide incidence (7/100,000), suggesting unique environmental/genetic factors involved in esophageal and gastric cardia cancer in the Chaoshan area [10, 11]. In the present study, we collected surgical specimens of esophageal and gastric cardia NEC patients who underwent esophagectomy with regional lymphadenectomy in Chaoshan Hospital from January 2007 to December 2016, and investigated the clinicopathologic and immunohistochemical data following tumor definition based on the WHO classification of NECs.

## RESULTS

All medical records of esophageal cancer and gastric cardia cancer patientsin the Chaoshan region from 2007 to 2016 were searched. Among 3105 esophageal cancer and 823 gastric cardia cancer patients, 67 (2.1%) esophageal NEC and 13 (1.6%) gastric cardia NEC cases were diagnosed and selected for further investigation.

### Clinical characteristics

The clinical characteristics of 67 esophageal patients from the Chaoshan area are summarized in Table 1. Of the 67 esophageal NEC patients, 47 were men and 20 were women, with the ratio of men to women being 2:1, and the average patient age was 58.5 years (ranging from 44 to 75). Overall, 54/67 (80.5%) of the NEC patients had dysphagia for over 20 days to 3months, and 11/67 (16.4%) patients felt pain in the chest. Gastroscopy, esophagoscopy or gastroesophageal CT was performed on 65 patients before pathological diagnosis. Just like esophageal squamous cell carcinoma, the gross appearances of NECs were mainly divided into four types: ulcerated (24/67), fungating (18/67), medullar (20/67) and stenotic(5/67). On the tumor surface, necrosis was found in 88% of the cases, and blood was seen in 20 (29.8%) cases. The tumor size was 3.5 cm on average, and ranged from 0.2 to 8 cm. For esophageal NEC, the majority of tumors (40/67) were located in the middle segment of the esophagus, 13/67 in the upper esophagus, and 14/67 in the distal esophagus.

**Table 1:** Clinical features of esophageal NECs

Pathologic Characteristics	Total						
		SCNC	LCNC	*P*-value	Mixed NEC	Pure NEC	*P*-value
**Age**							
**> 59**	53	51	2	0.19	15	38	0.74
**≤ 58**	14	12	2		5	9	
**Location**								
**Upper**	13	12	1	0.91	1	12	0.06
**Middle**	40	38	2		12	28	
**Distal**	14	13	1		7	7	
**Tumor size**								
**< 3.5 cm**	34	33	1	0.36	9	25	0.60
**> 3.5 cm**	33	30	3		11	22	
**Gross appearance**								
**Ulcerated**	24	23	1	0.79	5	19	0.53
**Fungating**	18	17	1		5	13	
**Medullary**	20	18	2		8	12	
**Stenosis**	5	5	0		2	3	
**Gender**								
**Male**	47	44	3	1	13	34	0.57
**Female**	20	19	1		7	13	

The detailed information of gastric cardia NEC patients (*n* = 13) is listed in Table 2. The age of the gastric cardia NEC patients ranged from 29 to 78, with 8 of the 13 gastric cardia NEC patients presenting with symptoms of gastroesophageal reflux, and 5/13 presenting vomit or blood in the stool. The gastric cardia NECs were all located within 2 cm from the gastroesophageal junction. The majority of tumors were ulcerated (9/13) and had sizes ranging from 0.5-5.5 cm.

**Table 2:** Clinicopathological data on patients diagnosed with gastric cardia NEC

NO.	Gender	Age	Tumor size	Gross type	Symptom	Histopathology (NECs with)	Stage	IHC-positive staining	Treatment (Surgery)	Follow-up
1	M	68	2 cm	ulcerating	GR	AD	T2N0M0 (I)	Syn, CK		NA
2	M	69	4.5 cm	fungal	VB	AD	T3N0M0 (II)	Syn, CgA, CEA		NA
3	M	65	0.5 cm	fungal	VB	AD	T3N0M0 (II)	Syn, CgA	CT	NA
4	M	63	2.3 cm	ulcerating	GR	MAD	T4N3M0 (III)	Syn, CK8/18		5
5	M	58	1.7 cm	ulcerating	VB	AD	T4N2M0 (III)	Syn, CgA		10
6	M	78	0.8 cm	ulcerating	GR	Pure	T3N1M0 (II)	Syn, CD56	CT	NA
7	M	44	1.1 cm	ulcerating	GR	Pure	T3N0M0 (II)	Syn, CD56		NA
8	M	65	3.5 cm	ulcerating	VB	AD	T4N0M0 (III)	Syn, CK8/18		NA
9	M	65	1.3 cm	ulcerating	GR	Pure	T3N1M0 (II)	Syn, CgA, CK		18
10	M	78	0.5 cm	fungal	GR	Pure	T4N0M0 (III)	Syn, CgA, CK	CT	NA
11	F	69	0.9 cm	fungal	GR	MAD	T3N1M0 (II)	Syn, CgA		NA
12	F	63	3.4 cm	ulcerating	VB	Pure	T4N0M0 (III)	Syn, CD56	CT	10
13	F	29	2.2 cm	ulcerating	GR	AD	T2N0M0 (I)	Syn, CK	CT+RT	Alive

M: male; F: female; GR: gastroesophageal reflux, VB: vomit or blood in the stool CT: chemotherapy; RT: radiotherapy

### Pathology stage

Based on the 8th edition of the AJCC TNM staging system for ESCC, the tumor stages of the esophageal NECs are shown in Table 3. A significant difference was observed in tumor differentiation and clinical stage between pure NEC and mixed NEC groups. The pathological features of gastric cardia NEC patients are listed in Table 2. Lymph node metastasis was found in 5/13 cases, with metastasis of the regional lymph node being the most common. The majority of cases (11/13) were stage II–III, and none of the cases displayed distant metastasis.

**Table 3:** Pathological stages of esophageal NEC patients

Pathologic Characteristics	Total	Classification					
		SCNC	LCNC	*p*	Mixed NEC	Pure NEC	*P*
Differentiation (G)							
Well (G1)	0	0	0	0.57	0	0	0.04^*^
Moderate (G2)	19	19	0		2	17	
Poor (G3)	48	44	4		18	30	
Invasion							
T1	3	3	0	0.50	2	1	0.26
T2	14	13	1		3	11	
T3	30	27	3		7	23	
T4	20	20	0		8	12	
Lymph node metastasis							
N0	43	40	3	0.57	17	26	0.07
N1	14	14	0		1	13	
N2	7	6	1		1	6	
N3	3	3	0		1	2	
Distal Metastasis							
M0	58	54	4	1	15	43	0.11
M1	9	9	0		5	4	
Clinical Stage							
I	17	17	0	0.22	8	9	0.02^*^
II	18	17	1		3	15	
III	21	18	3		3	18	
IV	11	11	0		6	5	

### Histopathologic features

**Figure 1:**
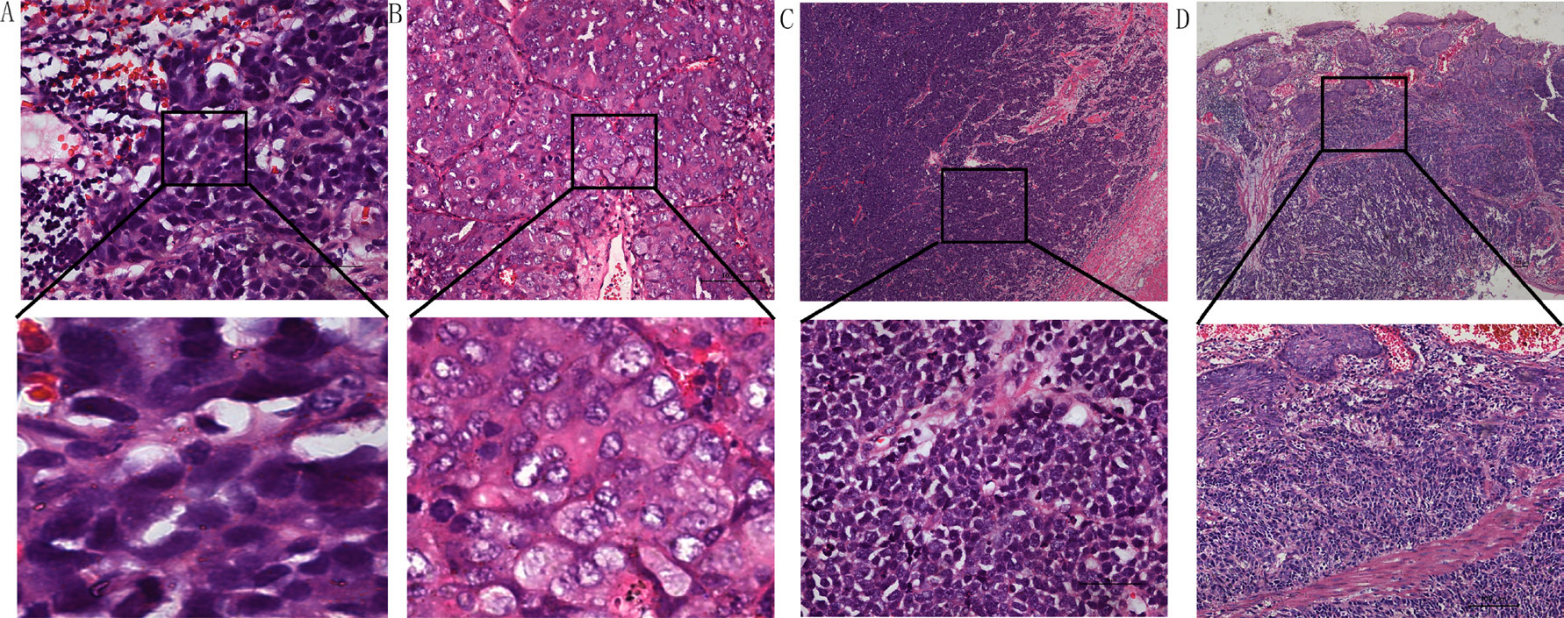
(**A**) Small cell esophageal NEC showing oat-like features. (**B**) Large cell esophageal NEC. (**C**) Pure esophageal NEC. (**D**) Mixture of NECs and squamous cell carcinoma. Hematoxylin-eosin stain in (A–D).(Scale bar: 100 μm)

As shown in Table 1, under high-power observation, the majority (94%) of esophageal NEC cells displayed a round/ovoid, spindle or anaplastic shape, indicating tumorscould be classified as small cell neuroendocrine carcinomas (SNECs) (Figure 1A). In contrast, large cell neuroendocrine carcinoma cells(LNECs) were large with irregular, bizarre nuclei and ample eosinophilic cytoplasm (Figure 1B). Esophageal SNEC was present in 63 (94.0%) patients, and 4 (6%) patients had LNEC; 20 (29.9%) patients had mixed NEC, and 47 (70.1%) patients had pure NEC. Two (10%) patients had mixed NEC and adenocarcinoma, and 18 (90%) patients had mixed NEC andsquamous carcinoma, including 17 (94.4%) patients with SNEC and 1 (0.6%) with LNEC (Figure 1C, 1D).

**Figure 2:**
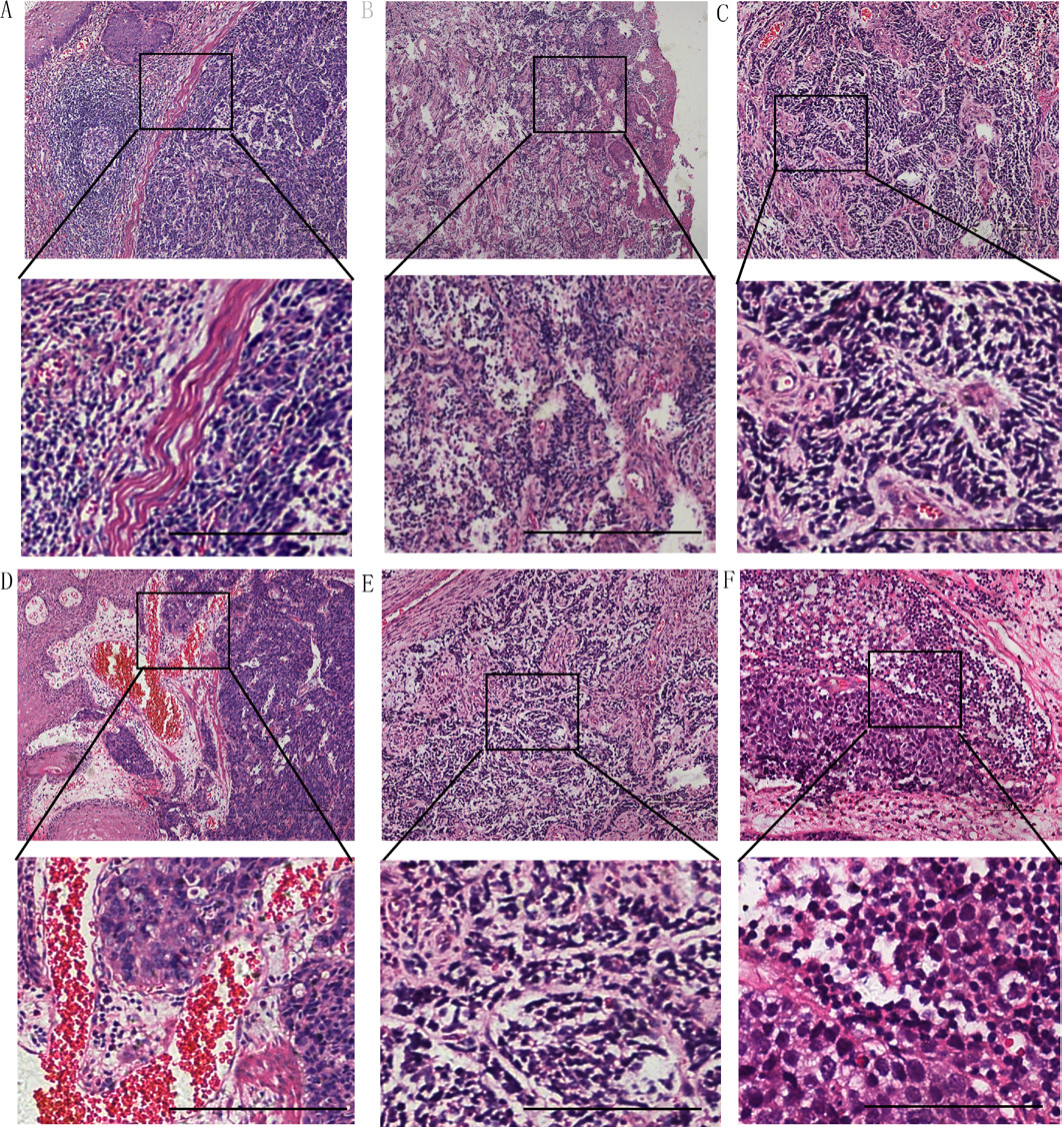
(**A**) The boundary between NEC and squamous cell carcinoma is clear and lymphocytes infiltrating into lymphoid follicles. (**B**) NEC cells were mixed with esophageal squamous carcinoma cells without a border. Tumor stroma was infused with blood vessels in most cases with capillaries (**C**) and venules (**D**). Lymph node metastasis could be found in 24 cases (**E**, **F**). Hematoxylin-eosin stain in A-D.(Scale bar: 100 μm)

Table 1 compares the clinicopathologic features of SNEC *vs.* LNEC, and pure NEC *vs.* mixed NEC. No significant difference was noted in age, sex, gross type, tumor location or tumor size between these groups. Microscopic observation revealed that the majority of NECs exhibited a nodular growth pattern with a clear border. The tumor border was infiltrated with capillaries and inflammatory cells, such as lymphocytes and plasmacytes, and two tumors showed rare lymphoid follicles (Figure 2A). However, some tumors displayed an invasive pattern in that the tumor cells broke the tumor boundary to mix with other types of cancer cells or normal cells (Figure 2B). Tumor stroma was rich in vasculature in most (83.6%) cases with marked proliferation of capillaries and small venules both being most prominent in the superficial portion of the tumor, and with submucosal and subserosal regions surrounding the tumor (Figure 2C, 2D). In all esophageal NEC tumors, intratumoral small lymphocytic infiltration was characteristically absent or minimal. Calcification, osteoid metaplasia, or granuloma was not observed in any cases. Tumor metastasis to regional nodes (24/24) and abdominal celiac lymph nodes (11/24) was substantial(Figure 2E, 2F). The data further showed that metastasis affected the juxta-esophageal lymph node (11/24), trachea carina (8/24), left supraclavicular lymph nodes (7/24), left gastric arteria lymph node (4/24), left recurrent laryngeal nerve lymph node (3/24) and stomach cardia lymph node (2/24).

**Figure 3:**
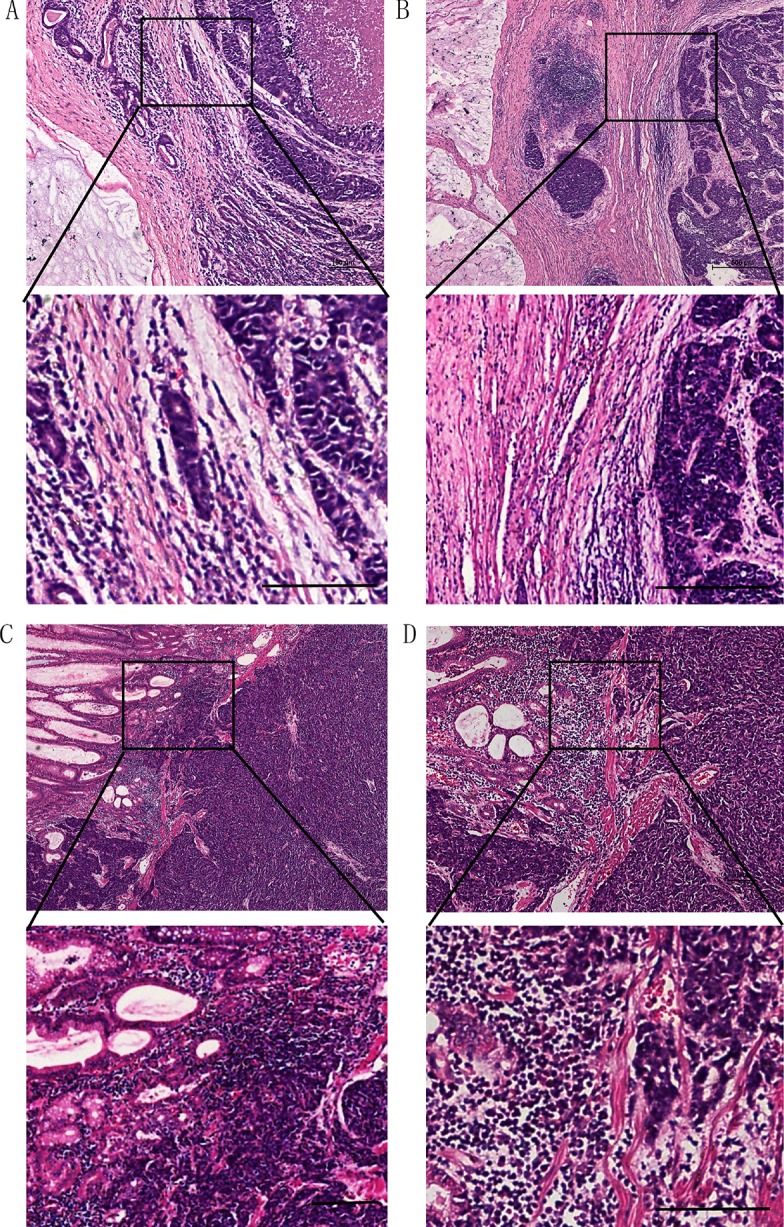
In gastric cardia NECs, some cases were mixed with mucinous adenocarcinoma, and with a clear border (A, B) Some were mixed with adenocarcinoma, and with a broken boundary (**C**, **D**).(Scale bar: 100 μm)

In gastric cardia NEC patients, all NEC swere of the small type, with 8/13 patients showing mixed NEC with adenocarcinoma. In particular, two NEC patients had a mixed NEC with mucinous adenocarcinoma. The border between these two types of cancer was clear and wide, and contained infiltrating inflammatory cells (Figure 3A, 3B). In some pure NEC cases, the cancer cells had a tendency to break the boundary and invade into the surrounding normal tissues (Figure 3C, 3D).

### Immunohistochemical studies

**Figure 4:**
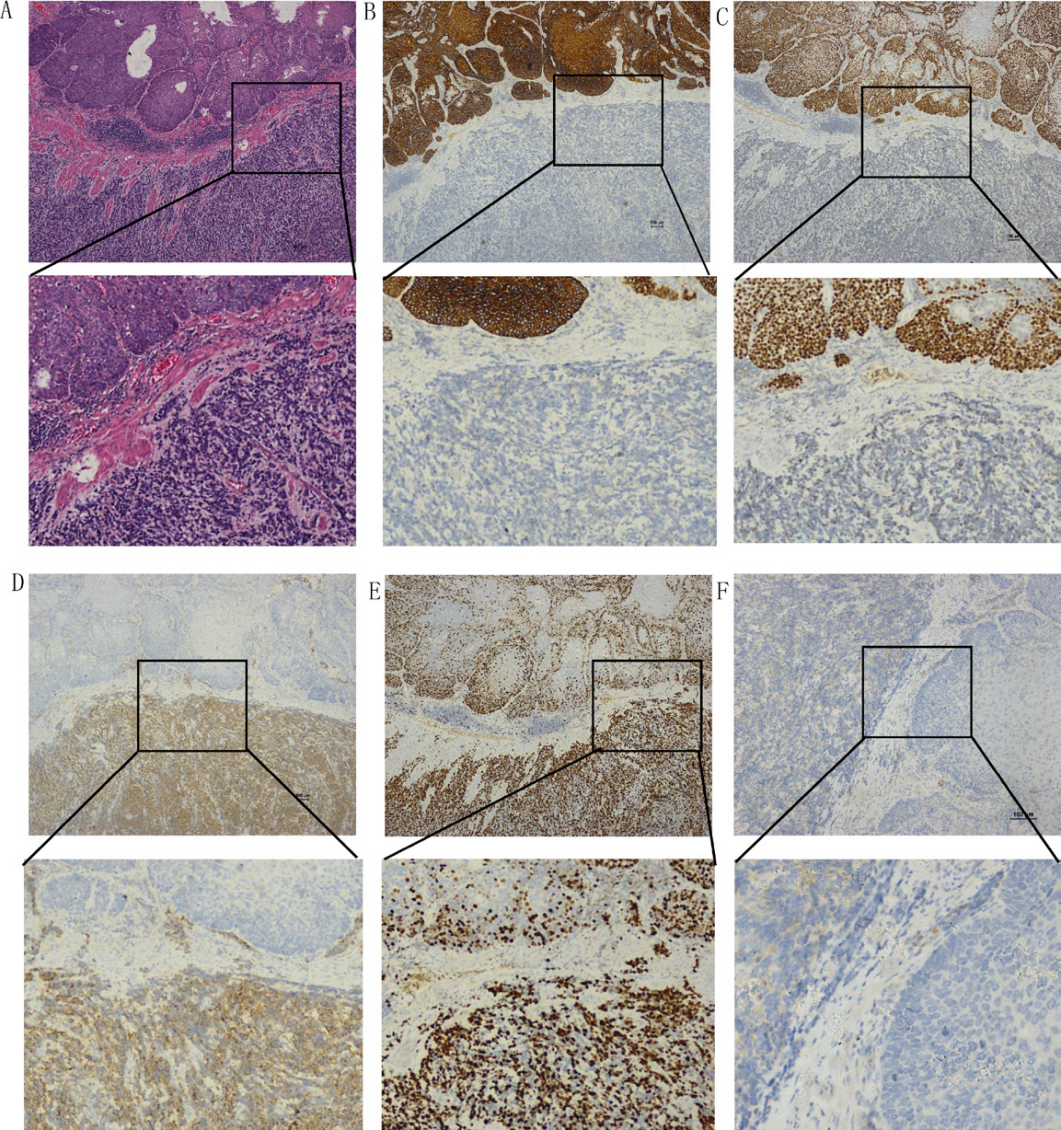
Examples of immunohistochemical staining of mixed type NEC and squamous carcinoma cells (**A**) Hematoxylin-eosin stain. Immunoactivity to CK (**B**), P63 (**C**), Syn (**D**), Ki67 (**E**), and CgA (**F**).(Scale bar: 100 μm)

Syn, CgA, CD56, S-100, et al were used to detect the NECs (Figure 4). Esophageal neoplastic cells exhibited strong, diffuse immunoreactivity to Syn in all cases and to NSE in 9/10 cases (Table 4). Strong immunoreactivity was also observed for CD56 in 18/20 of the cases, and to CgA in 30/54 cases, with variable proportions. Neoplastic cells in 7/14 of the cases were also immunoreactive for S-100. Positive nuclear TTF-1 immunoreactivity was demonstrated in 6/11 of the cases. In the mixed NEC patient samples, 7/20 tumors were immunoreactive to p63 antibody and 11/15 cases were positive for CK. Evident immunoreactivity to Ki67 was substantial (ranging from 30%–90%of the NEC cells). There was no significant difference in any of the immunoreactivities between large cell and small cell types (Table 4).

**Table 4:** Immunostaining for CgA, Syn, CD56, NSE, S100, P63, TTF-1, and CK

Antigen	Total	Classification		
		SCNC	LCNC	*P*
CgA	30/56	28/53	2/3	1
Syn	67/67	63/63	4/4	1
CD56	18/20	16/17	2/3	0.28
NSE	9/10	9/10	0	1
S100	7/14	6/13	1/2	1
P63	7/20	6/19	1/1	0.35
TTF-1	6/11	6/11	0	1
CK5/6	11/15	8/11	3/4	1

Immunohistochemistry of gastric cardia NEC tumors is listed in Table 2. Syn was positive in 7/7 cases, and 6/6 cases were positive for CgA. Immunoreactivity was also observed for CK5/6 in 4/5 of the cases and the positive incidence of Ki67 ranged from less than 30% to 70%.

### Patient survival

Survival data was available for 34/67 patients, and the overall median follow-up period was 23.0 months, within which 17 patients died. The 1–, 3– and 5-year overall survival was 88.2%, 55.9% and 29.5%, respectively, with no significant difference in the survival of NEC and ESCC patients.

Univariate and multivariate analyses were performed to assess the relationship between clinicopathological features and prognosis (Table 5). In univariate analysis, lymph node metastasis and distant metastasis showed statistical difference. Survival of patients with lymph node metastasis was significantly shorter (24.5 vs. 10.7 months, *P* = 0.02). Patients with distant metastasis revealed a shorter survival time than patients without (11.6 vs. 33.8 months, respectively, *P* = 0.01). However, univariate analysis showed there were no significant differences in survival time with regard to gender (*P* = 0.74), age (*P* = 0.79), tumor site (*P* = 0.62), gross tumor type (*P* = 0.44), tumor invasion (*P* = 0.26), tumor differentiation (*P* = 0.21), tumor size (*P* = 0.98) and having undergone or not undergone chemotherapy (*P* = 0.48). Furthermore, in multivariate analysis conducted using the Cox proportional hazards mode, lymph node and distant metastasis showed significant differences (*P* = 0.01), with patients with lymph node or distant metastasis having a shorter median survival time.

**Table 5:** Univariate and multivariate analysis of prognostic factors for esophageal NEC

	Univariate		Multivariate	
	HR (95%CI)	*P*-value	HR (95%CI)	*P*-value
**Age, years (< 58 vs. ≥ 58)**	1.3 (0.1–15.2)	0.79		
**Gender (male vs. female)**	0.75 (0.39–1.44)	0.74		
**Tumor site (middle vs. others)**	1.34 (0.25–7.89)	0.62		
**Tumor size (> 5cm vs.< 5cm)**	1.05 (0.05–23.2)	0.98		
**Gross type (variable)**	0.33 (0.75–2.66)	0.44		
**Depth of invasion (continuous variable)**	0.51 (0.05–5.17)	0.26		
**Lymph node metastasis (yes vs. no)**	14.74 (1.26–172.4)	0.02	6.86 (2.22–21.21)	0.01
**Differentiation (continuous variable)**	0.11 (0.01–3.32)	0.21		
**Treatment (CT vs. no CT)**	0.37 (2.96–6.75)	0.48		
**Distant Metastasis (yes vs. no)**	33.1 (2.02–543.5)	0.01	15.6 (3.14–77.62)	0.01

## DISCUSSION

In this paper, we characterize the clinicopathological features of neuroendocrine carcinoma of the esophagus and gastric cardia, a relatively rare malignant tumor, in the Chaoshan region. The characteristics of esophageal NECs are as follows:1. tumors are predominantly located in the center of the esophagus; 2. the small cell type comprises the main histological classification; 3. gross appearance is mainly of the ulcerative type; 4. half of the patients have lymph node metastasis and the juxta-esophageal lymph node is the most frequently affected; and 5. patients who have distal metastasis or lymph node metastasis have a worse clinical outcome. Gastric cardia NEC is primarily mixed with adenocarcinoma, the ulcerative type is the most common gross appearance, and the regional lymph node is the most frequent metastatic site.

NEC is defined as a high grade neuroendocrine carcinoma (G3), data regarding the prevalence of esophageal and gastric cardia NEC among patients is limited. Lyomasa et al. reported that esophageal NECs comprise approximately 1–2% of all esophageal neoplasms in China [13]. The current study indicates that the incidence of esophageal NECs has been approximately 2.1% of all esophageal cancers in Chaoshan over the past 6 years. Esophageal NEC closely correlates with Barrett's esophagus [14]. However, the origin of this tumor has not been clearly demonstrated. Some reports indicate that NEC originates from Kulchisky cells in the esophageal epithelium, which are amine precursor uptake and decarboxylation (APUD) cells, since theyhave APUD characteristics [15]. However, other reports indicate esophageal NECs originate from pluripotent stem cells that can differentiate into various epithelial cells. Our finding supports this concept since NECs are always mixed with other types of cancer, e.g. SCC or adenocarcinoma, especially in the gastric cardia.

In general, esophageal cancers are often present as an intraluminal exophytic mass, which results in an early onset of symptoms and allows for earlier diagnosis and treatment. Although esophageal NECs have a tendency for endoluminal growth, this does not mean that this type of tumor has limited malignant potential. We found that the major gross type of NEC is ulcerated, with the necrosis and hemorrhage on the surface of the tumor. In the majority of cases, cancer cells have infiltrated into the muscular layer as far as the serosa layer, indicating that NEC cells can be aggressively invasive.

The major pathological type of esophageal NEC involves small cell NEC, with most esophageal NECs are pure NECs. We only observed two NECs mixed with adenocarcinoma and others mixed with SCC. In those two cases, the patients had a history of gastrointestinal reflux. Our findings are consistent with Huang et al., who described 42 patients suffering from high-grade neuroendocrine carcinoma of the esophagus in China and found that most (88%) NECs were of the small cell type [13]. However, Maru et al. reviewed 40 cases of esophageal NEC reported in the USA, in which 27/40 cases were of the large cell type [16]. In contrast, our data show that an overwhelming number of NECs in the Chaoshan population is of the small cell type, suggesting that NEC in the Chinese population represents a unique esophageal neoplasm. One possible explanation of this difference is the geographical variation and population genetics.

Our group of esophageal NEC patients, in the Chaoshan region, were in the late stages, with 32 of the cases being in stage III–IV, 24/67 of patients presenting with metastasis, mainly of the regional lymph nodes, and 9 patients with distant metastasis. A high frequency of lymph node metastases and distant metastases has been reported for esophageal NECs. Recently, Nayal et al. reported 11 cases with small cell carcinoma of the esophagus in India, with 8/11 having distant metastases, the liver and lung being common sites [17, 18]. Huang et al. found that half of esophageal NEC cases have lymphovascular (50%) and lymph node metastasis [13]. In our study, all patients had surgical resection and lymphadenectomy. The literature reports that neuroendocrine carcinoma of the esophagus has lymph node metastasis in the very early stages, and that the ratio of lymph node metastasis is higher than for esophageal squamous cell carcinoma [15]. The importance of lymphadenectomy should be highlighted as much as possible, since this is a possible approach to cure the disease. Patients with lymphadenectomyhave good prognosis [15].

Although it is not essential to do immunohistochemistry to diagnose NEC tumors, IHC is very useful for identifying typical NEC histology and cell differentiation. Positive staining of neuroendocrine markers is an important supplement for the diagnosis of NEC. Generally, neuroendocrine biomarkers, such as Syn and CgA, are essential for the diagnosis of NECs. In our patient cohort, NEC tumors display 100% positive staining for Syn, and 53.6% positive staining for CgA, which is lower than a previous report [13]. Our findings are consistent with Huang et al., who reported that Syn staining is positive in all esophageal NECs [19].

There are limited reports concerning the outcome of patients with esophageal NEC, owing to the rarity of the disease. However, the prognosis for NEC is poor, since there is a high likelihood for disease recurrence. In this study, we performed Cox univariate and multivariate analysis and showed that tumor invasion and lymph node metastasis are independent factors for prognosis of NEC. The 1-and 3-year survival for esophageal NEC in our cases is 88.2 and 55.9%, respectively. The poor prognosis of esophageal NECs could be a result of early lymphatic spreading and distant metastasis. Some have reported that invasion depth is the key factor for determining the survival of NEC patients [20]. The major difference of patient prognosis is between T1 and T2–4, indicating that patients have poor prognosis once the tumor has invaded the esophageal muscular layer. Early diagnosis and treatment are also important in that surgery has a limited effect, although joint postoperative adjuvant radiation and chemotherapy can obviously improve prognosis. Survival analyses by Lu et al. [21] and Ding et al. [22] reveal that joint treatment of patients is better than surgery only. Patients with mixed tumors have better prognosis than patients with pure NEC. However, there is no difference in prognosis between the large cell type and small cell type. A previous report stated that the prognosis of mixed NEC patients is worse compared with patients with pure NECs, but other reports indicate that the survival of mixed NEC patients and pure NEC is not obviously different [23].

In our study, we found that gastric cardia NECs are mostly mixed with high grade adenocarcinoma, and the borders are surrounded by loose connective tissue with inflammatory cells. Reports of gastric cardia NEC are very limited because of the unique physiological role of this mucosa. Some researchers found that neuroendocrine cells of gastric cardia are present in Barrett's esophagus, a severe gastro-esophageal reflux disease [24]. Voutilainen et al. reported 18 CgA-positive gastric cardia cases. However, they did not state the stage of the NETs. Most NETs in gastric cardia are of low-intermediate grade, and that among 76 cases of gastric cardia with NETs, only 11 cases involved NEC, which had a larger tumor size and deeper invasion [8].

Our paper studies 80 cases of esophageal and gastric cardia NEC, in the Chaoshan area, and characterizes the clinicopathological and immunohistochemical features, which may provide some therapeutic clue for treating this type of rare tumor.

## MATERIALS AND METHODS

### Patient sample collection

We collected and reviewed cases of patients diagnosed with primary esophageal and gastric cardia NEC from 2007 to 2016 in the Chaoshan area. The electronic medical records of all patients with esophageal and gastric cardia cancer, including the gender, age, tumor location, size, gross appearance, and histologic features, were searched in the Department of Pathology of the Shantou Tumor Hospital, First Affiliated Hospital of Shantou University Medical College and Shantou Central Hospital. All cases were independently reviewed and diagnosed by two pathologists. The study was approved by the ethical review committees of the Medical College of Shantou University. All participants involved in our study gave written informed consent. Retrospective collection of NECs had been approved by each ethical review committee of all participating centers. All experiments performed in this paper were in accordance with the relevant guidelines and regulations [1].

The inclusion criteria of case selection were as follows: 1. diagnosis of the pathology, along with the medical, radiologic, and endoscopic records, confirmed NEC of the esophagus and gastric cardia; 2. all clinical parameters, including the age, gender, and symptoms, were detailed in the records; 3. neoplastic cells in the cases were immunoreactive to the WHO-recommended neuroendocrine markers, such as CD56, synaptophysin (Syn), and chromogranin A (CgA); 4. the center of the esophageal NEC tumor was located within 2 cm above the gastroesophageal junction (GEJ), defined by the American Joint Cancer Committee, eighth edition, for esophageal cancer, and the center of gastric cardia NEC tumors was located > 2 cm from the GEJ.

Exclusion criteria included the following: 1. the patient had prior chemotherapy or radiation treatment before surgical resection; 2. clinical data were not available, even if the cancer was diagnosed asesophageal or gastric cardia NEC; 3. the patient had a history ofneuroendocrine carcinoma elsewhere.

### Histopathologic assessment

NEC patient age, gender, symptoms, location, gross appearance, tumor size, clinical stage, classification, invasion, and lymph node metastasis, immunohistochemical expression, lymphovascular invasion, perineural invasion, radiologic findings, types of treatment and response to treatment were all collected. The location of the esophageal NEC was dividedinto three parts: upper (15–24 cm from the incisorteeth), middle (25–32 cm from the incisor teeth) and lower (33–40 cm from the incisor teeth). The location of gastric cardia NEC was a narrow band between the lower esophagus and gastric fundus that was limited to within 2 cm from the GEJ. Tumors located between the distal esophagus and gastric cardia were carefully evaluated based on the location and the adjacent mucosa. The gross appearance of esophageal and gastric cardia NEC was mainly categorized into 4 types: ulcerated, fungating, medullar and protuberant. Regional lymph nodeor distant metastasis was mainly evaluated using imagingmodalities, including CT and endoscopic ultrasonography (EUS), as well asother modalities. Lymph nodemetastasis was confirmed by apost-operative pathologic report if the imageological examination was uncertain. The locations of lymph node metastasis were also recorded. Results of lymphovascular invasion, perineural invasion and lymph nodemetastasis were presented as either positive or negative. The clinical stage of NECs followed the rules for ESCC as defined by the American Joint Cancer Committee in the eighth edition of the esophageal cancer staging manual, since standardized staging guidelines for NEC are lacking.

NEC was classified as small cell NEC (SNEC) and large cell NEC (LNEC) according to the WHO criteria for NEC [12]. SNEC cells are 2–3 times larger than lymphocytes, with scant cytoplasm and an inconspicuous nucleolus. However, LNEC cells are more than 4 times larger than lymphocytes, with ample cytoplasm and an evident nucleolus. Both SNEC and LNEC have and frequent necrosis and high mitotic rate (> 20/high power field), with positive immunostaining for neuroendocrine markers. NEC can also be classified into 3 groups based on differentiation according to the 2000 WHO classificationcriteria: well-differentiated, moderately-differentiated and poorly-differentiated NEC.

### Immunohistochemistry and evaluation

Immunohistochemical staining was performed using routine methods [13]. Images were captured using a Leica IM50 microscope (Imagic Bildverarbeitung AG, Wetzlar, Germany) at × 100, × 200 and × 400. IHC staining was examined by two pathologists, who were blinded to the clinical outcome. Tumors were defined as positive if over 30% of neoplastic cells stained positively for the antigen. Staining was determined by consensus andthe discrepancy between readers was minimal.

Primary antibodies, CD56 (1:200), Syn (1:200), CgA (1:200), cytokeratin 5/6 (CK 5/6) (1:200), p63 (1:100), TTF-1 (1:200), smooth muscle actin (SMA) (1:200), Ki67 (1:500) and S-100 (1:200) were purchased from Maixin (China). Immunohistochemical staining results were presented as either positive or negative.

### Survival outcome

We performed the final follow-up by telephone, mail or outpatient department visit in October 2016. Overall survival was calculated from the day of esophagogastroduodenoscopy and biopsy to thedate of death or last follow-up (months). Clinical follow-up data was available for 34/67 esophageal NEC patients and 3/13 of gastric cardia NEC patients included in this study.

### Statistical analysis

Statistical analysis was performed using SPSS software (SPSS Corp, Chicago, IL, USA). Data are represented asthe mean ± standard deviation for continuous variables or number (%) for categorical data. To estimate the association between eligible variables and mean survival time, Kaplan-Meier analysis was applied. A *p*-value less than0.05 was considered statistically significant.

### Ethics approval and consent to participate

The study was approved by the ethical review committees of the Medical College of Shantou University. The patients involved in our study provided written informed consents.

### Consent for publication

We received permission from the patients to publish the data.
